# Association of the 2020 US Presidential Election With Hospitalizations for Acute Cardiovascular Conditions

**DOI:** 10.1001/jamanetworkopen.2022.8031

**Published:** 2022-04-20

**Authors:** Matthew T. Mefford, Jamal S. Rana, Kristi Reynolds, Omesh Ranasinghe, Murray A. Mittleman, Jennifer Y. Liu, Lei Qian, Hui Zhou, Teresa N. Harrison, Alan C. Geller, Richard P. Sloan, Elizabeth Mostofsky, David R. Williams, Stephen Sidney

**Affiliations:** 1 Department of Research and Evaluation, Kaiser Permanente Southern California, Pasadena; 2 Department of Cardiology, Kaiser Permanente Oakland Medical Center, Oakland, California; 3 Division of Research, Kaiser Permanente Northern California, Oakland; 4 Department of Health Systems Science, Kaiser Permanente Bernard J. Tyson School of Medicine, Pasadena, California; 5 Department of Epidemiology, Harvard T.H. Chan School of Public Health, Boston, Massachusetts; 6 Cardiovascular Division, Department of Medicine, Beth Israel Deaconess Medical Center, Boston, Massachusetts; 7 Department of Social and Behavioral Sciences, Harvard T.H. Chan School of Public Health, Boston, Massachusetts; 8 Department of Psychiatry, Columbia University Irving Medical Center, New York, New York; 9 Department of African and African American Studies, Harvard University, Cambridge, Massachusetts

## Abstract

**Importance:**

Prior studies found a higher risk of acute cardiovascular disease (CVD) around population-wide psychosocial or environmental stressors. Less is known about acute CVD risk in relation to political events.

**Objective:**

To examine acute CVD hospitalizations following the 2020 presidential election.

**Design, Setting, and Participants:**

This retrospective cohort study examined acute CVD hospitalizations following the 2020 presidential election. Participants were adult members aged 18 years or older at Kaiser Permanente Southern California and Kaiser Permanente Northern California, 2 large, integrated health care delivery systems. Statistical analysis was performed from March to July 2021.

**Exposure:**

2020 US presidential election.

**Main Outcomes and Measures:**

Hospitalizations for acute CVD around the 2020 presidential election were examined. CVD was defined as hospitalizations for acute myocardial infarction (AMI), heart failure (HF), or stroke. Rate ratios (RR) and 95% CIs were calculated comparing rates of CVD hospitalization in the 5 days following the 2020 election with the same 5-day period 2 weeks prior.

**Results:**

Among 6 396 830 adults (3 970 077 [62.1%] aged 18 to 54 years; 3 422 479 [53.5%] female; 1 083 128 [16.9%] Asian/Pacific Islander, 2 101 367 [32.9%] Hispanic, and 2 641 897 [41.3%] White), rates of hospitalization for CVD following the election (666 hospitalizations; rate = 760.5 per 100 000 person-years [PY]) were 1.17 times higher (95% CI, 1.05-1.31) compared with the same 5-day period 2 weeks prior (569 hospitalizations; rate = 648.0 per 100 000 PY). Rates of AMI were significantly higher following the election (RR, 1.42; 95% CI, 1.13-1.79). No significant difference was found for stroke (RR, 1.02; 95% CI, 0.86-1.21) or HF (RR, 1.18; 95% CI, 0.98-1.42).

**Conclusions and Relevance:**

Higher rates of acute CVD hospitalization were observed following the 2020 presidential election. Awareness of the heightened risk of CVD and strategies to mitigate risk during notable political events are needed.

## Key Points

**Question:**

Did acute cardiovascular disease hospitalizations increase immediately after the 2020 presidential election?

**Findings:**

In this cohort study of 6 396 830 adults, the rate of hospitalization for acute cardiovascular disease was 17% higher in the 5 days following the election compared with the same 5-day period 2 weeks prior. The rate of acute myocardial infarction was 42% higher, but no significant difference was found for heart failure or stroke.

**Meaning:**

These findings suggest that awareness of the heightened risk of cardiovascular disease and strategies to mitigate risk during notable political events are needed.

## Teaser

This cohort study examines acute cardiovascular disease (CVD) hospitalizations following the 2020 US presidential election.

## Introduction

Prior studies have found a higher risk of acute cardiovascular disease (CVD) events immediately following behavioral, psychosocial, and environmental triggers.^1,2,3^ However, less is known about the affect of political events such as presidential elections on acute CVD risk. In a 2017 survey by the American Psychological Association, more than half (52%) of the respondents noted the current political climate as a substantial source of stress, and approximately two-thirds of the respondents noted that concerns about the future of the nation were a substantial source of stress.^4^ An update of the same survey conducted in August 2020 found a larger proportion of respondents (77%) indicating the future of the country as a substantial source of stress, enhanced by the ongoing COVID-19 pandemic.^5^ Furthermore, 68% reported the 2020 presidential election as a substantial source of stress, and this was high regardless of political party affiliation.^5^

In our previous study of Kaiser Permanente Southern California (KPSC) members, we found a 62% higher risk of hospitalization for acute myocardial infarction (AMI) or stroke in the 2 days immediately following the 2016 presidential election compared with the same 2 days in the 1 and 2 weeks prior.^6^ Other studies have similarly presented evidence of cardiovascular and noncardiovascular adverse outcomes associated with political stress and, more specifically, the 2016 presidential election.^7,8,9^ The aim of the current study was to replicate and expand on findings from our prior study of the 2016 presidential election that was limited to the Southern California population by examining hospitalizations for acute CVD around the 2020 presidential election among adults in KPSC and Kaiser Permanente Northern California (KPNC), 2 large integrated health care delivery systems that provide comprehensive care for more than 9 million persons.

## Methods

### Study Population

KPSC is an integrated health care delivery system with approximately 4.7 million members comprising more than 20% of the Southern California population. KPNC has a service area that encompasses the San Francisco Bay Area and the Central Valley from the Sacramento area in the north to Fresno in the south. KPNC provides care to approximately 4.5 million members comprising more than 30% of the population in its service area. The membership is highly representative of both Southern and Northern California populations with respect to age, sex, race and ethnicity, and socioeconomic status.^10,11^ For the current study, we included data from active members across both regions who were at least 18 years of age at the time of hospitalization. This study was approved by the institutional review boards at KPSC and KPNC, and waivers of informed consent were obtained owing to the data-only nature of this study. This study followed the Strengthening the Reporting of Observational Studies in Epidemiology (STROBE) reporting guideline.^12^

Age, sex, and race and ethnicity were obtained from patient electronic health records. Race and ethnicity were categorized into groups including Hispanic (regardless of race) and non-Hispanic racial groups including Asian/Pacific Islander, Black, White, and others; members with unknown race and ethnicity were excluded from race- and ethnicity-specific analyses. We included data for race and ethnicity to get an idea of the demographic breakdown of the population on the date of the election and to examine any possible heterogeneity by race and ethnicity (in addition to by age group and sex). The outcome of interest was hospitalization for acute CVD, defined as an inpatient principal discharge diagnosis of AMI (*International Statistical Classification of Diseases, Tenth Revision, Clinical Modification [ICD-10-CM]* codes I21.x and I22.x), an inpatient principal discharge diagnosis or emergency department principal discharge diagnosis of stroke (*ICD-10-CM* codes I60.9; I61.x-I63.x),^13^ or an inpatient principal discharge diagnosis of heart failure (HF) (ICD-10-CM codes I50.x, I11.0, I13.0, I13.2, I97.13, I97.130, I97.131, I09.81).^14^ The date of hospitalization was based on the time of admission. If an individual experienced multiple events of the same type within 7 days of the index event, they were counted as the same event. Secondary outcomes of interest were AMI, stroke, and HF, separately.

### Statistical Analysis

Daily rates of CVD hospitalization were calculated 1 month before and 1 month after the election date as the number of events per 100 000 person-years (PY). A smoothing trend of daily event rates using a 7-day moving average was used for descriptive purposes. The 7-day moving average was calculated by taking the arithmetic mean of daily event rates over 7 days before the date under observation. The 2020 US presidential election was held on November 3, and on November 7 most national media organizations projected the winner of the election. Accordingly, we chose 5 days following the election (November 4-8) as the hazard period. To minimize confounding due to intense traditional and social media coverage leading up to the day of the election, the week leading up to the election date was considered as a buffer period. Instead, the same 5-day period 2 weeks prior (October 21-25) was used as the control period. Using Poisson regression, rate ratios (RR) and 95% CIs were calculated to compare rates of hospitalization for acute CVD per 100 000 PY in the hazard period with the control period. In a sensitivity analysis, we used the same 5-day period in the 2 and 3 weeks before the election date (October 14-18 plus October 21-25, 10 days total). RRs were calculated for the entire cohort, and we examined associations within subgroups by age (18-54 years, 55-74 years, ≥75 years), sex, and race and ethnicity, testing for effect measure modification with Wald tests for heterogeneity. In an additional sensitivity analysis, we repeated the aforementioned analyses excluding patients with laboratory-confirmed or diagnosed COVID-19 during their hospitalization.

All analyses were repeated for AMI, stroke, and HF, separately. Statistical tests were 2-sided with a *P* < .05 considered statistically significant. Analyses were conducted using SAS statistical software version 9.4 (SAS Institute) from March to July 2021, and data were aggregated by KPSC.

## Results

**Figure 1.**
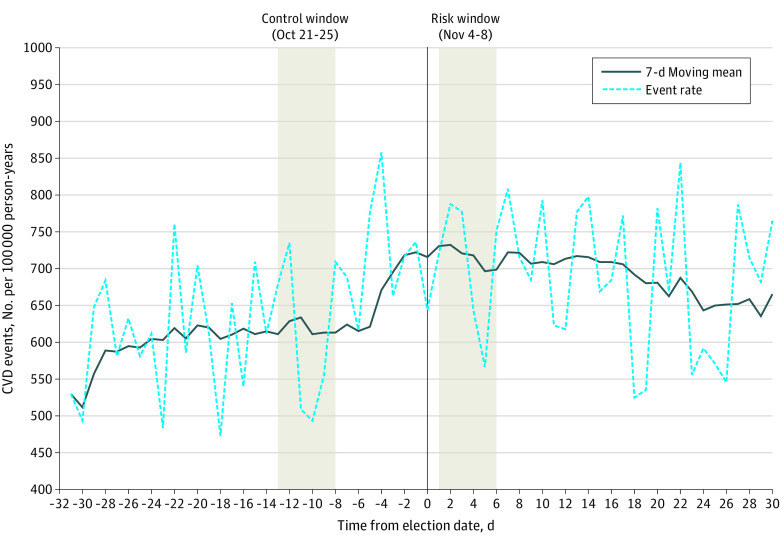
Hospitalization for Acute Cardiovascular Disease (CVD) Events per 100 000 Person-Years in the Month Preceding and After the 2020 Presidential Election Rates of composite cardiovascular disease events per 100 000 person years are presented 30 days before and after the date of the 2020 presidential election (0 = date of the 2020 presidential election). Absolute event rates are presented by a dashed line, with a 7-day smoothing average presented as a solid line. Rates in the risk window (November 4-8) and the control window (October 21-25) are highlighted.

Among 6 396 830 active KPSC and KPNC members who were at least 18 years of age on the date of the 2020 presidential election, 3 970 077 (62.1%) were aged 18 to 54 years, 1 896 302 (29.6%) were aged 55 to 74 years, and 530 451 (8.3%) were aged at least 75 years; 3 422 479 (53.5%) were female; 1 083 128 (16.9%) were Asian/Pacific Islander, 505 633 (7.9%) were Black, 2 101 367 (32.9%) were Hispanic, 2 641 897 (41.3%) were White, and 64 805 (1.0%) were other race and ethnicity categories. (Table 1). Daily rates of CVD hospitalizations 1 month before and after the 2020 election date with a moving average are presented in Figure 1. Rates of hospitalization for CVD in the 5 days following the 2020 presidential election (666 hospitalizations; rate = 760.5 per 100 000 PY) were 1.17 times higher (95% CI, 1.05-1.31) compared with the same 5-day period 2 weeks before the election (569 hospitalizations; rate = 648.0 per 100 000 PY). (Table 2) In subgroup analyses, rates of CVD hospitalization in the 5 days following the 2020 election compared with the same 5-day period 2 weeks before were higher among adults aged at least 75 years (301 vs 230 hospitalizations; RR, 1.31; 95% CI, 1.10-1.56; *P* for interaction by age group = .10), men (388 vs 239 hospitalizations; RR, 1.30; 95% CI, 1.12-1.51; *P* for interaction by sex = .04), and White individuals (336 vs 262 hospitalizations; RR, 1.29; 95% CI, 1.10-1.51; *P* for interaction by race and ethnicity = .51).

**Table 1.** Demographic Characteristics of Kaiser Permanente Southern and Northern California Adult Members, November 3, 2020

Characteristic	No. (%) (N = 6 396 830)
Age, y	
18-54	3 970 077 (62.1)
55-74	1 896 302 (29.6)
≥75	530 451 (8.3)
Sex	
Male	2 974 351 (46.5)
Female	3 422 479 (53.5)
Race and ethnicity	
Asian/Pacific Islander	1 083 128 (16.9)
Black	505 633 (7.9)
Hispanic	2 101 367 (32.9)
White	2 641 897 (41.3)
Other^a^	64 805 (1.0)

^a^ Other race and ethnicity includes Native American Alaskan, multiple ethnicities, and those not specified.

**Table 2.** Hospitalization for Acute Cardiovascular Disease Events in the 5 Days Following the 2020 Presidential Election (November 4-8) vs the Week Before the Election (October 21-25)

Characteristic	November 4-8 (risk window)			October 21-25 (control window)			Rate ratio (95% CI)	*P* value
	Events, No.	Person-years	Rate per 100 000 person-years	Events, No.	Person-years	Rate per 100 000 person-years		
Overall	666	87 577.82	760.47	569	87 812.46	647.97	1.17 (1.05-1.31)	
Age, y								
18-54	87	54 345.96	160.09	67	54 533.92	122.86	1.30 (0.94-1.79)	.10
55-74	278	25 964.30	1070.7	272	26 000.44	1046.14	1.02 (0.87-1.21)	
≥75	301	7267.56	4141.69	230	7278.10	3160.17	1.31 (1.10-1.56)	
Sex								
Male	388	40 721.97	952.80	299	40 848.65	731.97	1.30 (1.12-1.51)	.04
Female	278	46 855.85	593.31	270	46 963.81	574.91	1.03 (0.87-1.22)	
Race and ethnicity								
Asian/Pacific Islander	87	14 828.56	586.71	85	14 854.03	572.24	1.02 (0.76-1.38)	.51
Black	89	6922.46	1285.67	84	6939.75	1210.42	1.06 (0.78-1.43)	
Hispanic	150	28 771.25	521.35	132	28 826.69	457.91	1.14 (0.90-1.44)	
White	336	36 168.29	928.99	262	36 302.09	721.72	1.29 (1.10-1.51)	
Other^a^	4	887.27	450.82	6	889.91	674.23	NA	

Abbreviation: NA, not available.

^a^ Other race and ethnicity includes Native American Alaskan, multiple ethnicities, and those not specified.

**Figure 2.**
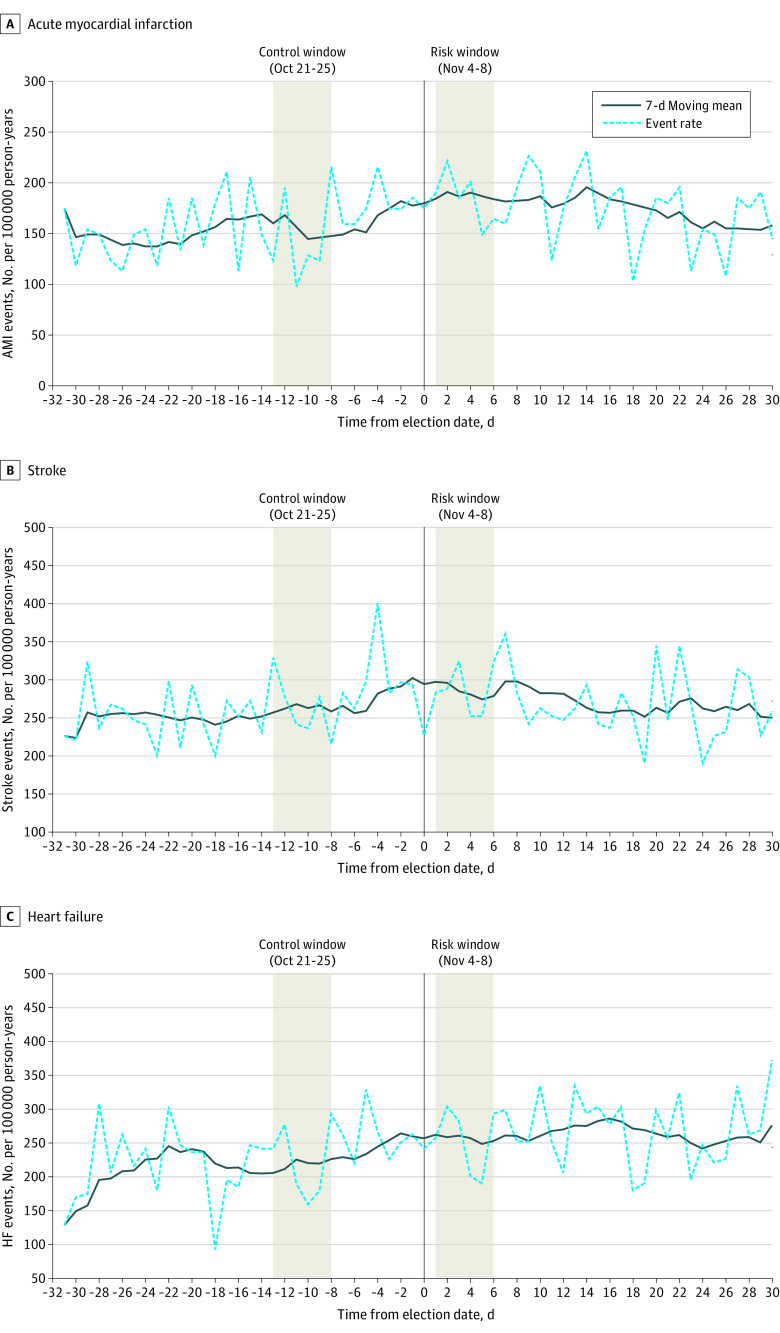
Hospitalization for Acute Myocardial Infarction (AMI), Stroke, and Heart Failure (HF) Events per 100 000 Person-Years in the Month Preceding and After the 2020 Presidential Election Rates of AMI (panel A), stroke (panel B), and HF (panel C) per 100 000 person years are presented 30 days before and after the date of the 2020 presidential election (0 = date of the 2020 presidential election). Absolute event rates are presented by a dashed line, with a 7-day smoothing average presented as a solid line. Rates in the risk window (November 4-8) and the control window (October 21-25) are highlighted.

Daily rates of AMI, stroke, and HF hospitalizations 1 month before and after the 2020 election date with a moving average are presented in Figure 2. Rates of hospitalization for AMI in the 5 days following the 2020 presidential election (179 hospitalizations; rate = 204.4 per 100,00 PY) were 1.42 times higher (95% CI, 1.13-1.79) compared with the same 5-day period 2 weeks before the election (126 hospitalizations; rate = 143.5 per 100 000 PY) (Table 3). Rates of stroke and HF were not higher during the 5 days following the 2020 presidential election compared with the same 5-day period in the 2 weeks prior (RR, 1.02; 95% CI, 0.86-1.21; and RR, 1.18; 95% CI, 0.98-1.42, respectively). There was no evidence of heterogeneity in the RR for CVD, AMI, stroke, or HF between KPSC and KPNC, with all *P* values for interaction by site greater than .05 (eTable 1 in the Supplement).

**Table 3.** Hospitalization for AMI, Stroke, and Heart Failure Following the 2020 Presidential Election (November 4-8) vs the Week Before the Election (October 21-25)

Condition	November 4-8 (risk window)			October 21-25 (control window)			Rate ratio (95% CI)
	Events, No.	Person-years	Rate per 100 000 person-years	Events, No.	Person-years	Rate per 100 000 person-years	
AMI	179	87 577.82	204.39	126	87 812.46	143.49	1.42 (1.13-1.79)
Stroke	265	87 577.82	302.59	261	87 812.46	297.22	1.02 (0.86-1.21)
Heart failure	239	87 577.82	272.90	203	87 812.46	231.17	1.18 (0.98-1.42)

Abbreviation: AMI, acute myocardial infarction.

In a sensitivity analysis, results were similarly unchanged when using an expanded control window of the same 5-day period in the 2 and 3 weeks before the election date for composite CVD hospitalizations and for AMI, stroke, and HF, separately (eTable 2 and eTable 3 in the Supplement). In an additional sensitivity analysis excluding patients with laboratory-confirmed or diagnosed COVID-19 during the same hospitalization (November 4-8: 12 patients; October 21-25: 8 patients), results for CVD hospitalizations were unchanged overall (RR, 1.17; 95% 1.04-1.31) and within age groups (RR, 1.31 [95% CI, 1.10-1.56] for adults aged at least 75 years), sex (RR, 1.31 [95% CI, 1.12-1.51] for men), and race and ethnicity (RR, 1.28 [95% CI, 1.09-1.50] for White members) (eTable 4 in the Supplement). Results for AMI (RR, 1.44; 95% CI, 1.15-1.82), stroke (RR, 1.01; 95% CI, 0.85-1.20), and HF (RR, 1.18; 95% CI, 0.98-1.42), separately, were also consistent with the main analysis when removing patients with laboratory-confirmed or diagnosed COVID-19. (eTable 5 in the Supplement).

## Discussion

In this study of 6 396 830 active KPSC and KPNC members at least 18 years of age, we observed a higher rate of hospitalization for acute CVD and, separately, AMI in the 5 days following the 2020 presidential election compared with the same 5-day period 2 weeks prior. These data support our findings from the 2016 election and are strengthened by the inclusion of an additional geographic region in California and an additional CVD end point.

There is growing evidence that psychological health contributes to CVD risk.^15^ Negative emotional traits such as anger and hostility have been associated with new and recurring coronary heart disease (CHD), as well as premature development of CVD.^16,17^ In patients with HF, mental stress was associated with acute worsening of LV diastolic pressure and recent episodes of anger associated with worse resting LV diastolic pressure.^18^ Additionally, anxiety is often tied to CVD risk factors that accelerate atherosclerosis. In a meta-analysis of 46 studies, anxiety was associated with a 41% higher risk of CVD, including statistically significant associations with CHD, stroke, and HF.^19^ Biologically, emotional distress may lead to inflammation,^20^ autonomic dysfunction,^21^ inability to control underlying CVD risk factors,^22,23^ and weakened immune responses^24^ that can also increase CVD risk. Leading up to the 2020 election and through several days afterward, there were claims of widespread voter fraud, different timelines for counting and reporting mail-in and in-person ballots, shifting vote totals, and speculation that results would not be acknowledged by one of the presidential candidates. Although speculative, it is plausible these factors combined created an emotionally charged atmosphere across the US regardless of demographic or political affiliation.

In our prior study,^6^ we used a 2-day risk window based on existing literature of other types of psychosocial events. In the current study we chose a longer window a priori because the final result was not known for 5 days after the date of the 2020 election, and to account for the protracted nature of associated stress and eventual announcement of the results. Results from this study are consistent with other studies surrounding the 2016 election.^6,7^ Rosman and colleagues^7^ studied patients in 2 health care centers in North Carolina, and they found a 77% higher risk of cardiac arrhythmia in the 6 weeks after the 2016 election compared with a similar period before the election. Results were consistent by age group, sex, and race and ethnicity, and the authors also reported no differences by political affiliation or political concordance or discordance—although they note small sample sizes as the potential limitation to some of these findings. In the absence of investigating biological mechanisms precipitating these events, they suggested that acute mental stress and negative emotions can produce changes in cardiac electrophysiology, which may trigger arrhythmia.^25^ Similarly, it has been noted that stress and anger are sufficient to trigger acute ischemia which may be sufficient to cause CVD events.^1,2,26^

We found a suggestion of higher rates of acute CVD following the election for older adults, men, and White individuals that may require more investigation. Biologically, CVD risk is high among older adults, but this may not explain the transiently higher risk following the election. Men are both less likely than women to report physical or emotional symptoms of stress and to take fewer actions in managing their stress levels.^27^ Although political affiliation was not examined, we can only speculate the reasons behind the larger impact on older adults, men, and White individuals. Given cardiovascular complications associated with COVID-19 including inflammation and hypercoagulation^28^ and the increasing prevalence of COVID-19 cases in the latter half of 2020, we wanted to rule out the impact of COVID-19 on hospitalization in our analysis. Few patients hospitalized during our risk and control windows had an accompanying diagnosis or laboratory-confirmation of a COVID-19 infection, and exclusion of these events did not substantially change our findings.

A wide array of potential stressors resulting from the COVID-19 pandemic, including health care disruptions; economic destabilization; uncertainty about working conditions, childcare, and schooling; and general fears of contracting COVID-19 were prominent in 2020 and we cannot rule out the potential influence of these circumstances on increasing CVD risk. However, these stressors occurred over a much broader and prolonged period and are less likely to explain the transient risks observed in our defined risk and control windows that are in close proximity to the 2020 election. Severe weather events have the potential to affect events or presentation to the hospital for evaluation, although this was beyond our scope to examine. Other weather-related exposures such as wildfires and, subsequently, exposure to poor air quality can acutely affect CVD risk, although even large increases in air pollution would be expected to have a much weaker association than the observed associations in the current study.

Future studies designed to look at stress reduction interventions may be important for understanding the intersection of political events, associated stress, and acute CVD risk. Encouraging exercise and other healthy lifestyle activities that can improve stress levels are reasonable targets for intervention.^29^ More immediately, clinical visits for cardiovascular reasons may be an opportunity to assess psychological factors that can influence a patient’s health maintenance and encourage stress reduction strategies.^15^ Mindfulness, meditation, yoga, and other relaxation techniques have also been suggested as ways to reduce stress.^30,31,32^ A meta-analysis^31^ of 42 studies that included yoga and mindfulness-based stress reduction techniques reported associations with lower evening and waking cortisol levels, lower systolic blood pressure, resting heart rate, heart rate variability, fasting blood glucose, cholesterol, and low-density lipoprotein-cholesterol compared with active controls.

Strengths of the current study include the use of 2 large, diverse integrated health care delivery systems that represent a population of insured individuals across the state of California. Importantly, results were consistent before and after excluding patients with confirmed COVID-19 infection. Although there have been prior studies on the 2016 election,^6,7,8^ to our knowledge, this is the first to examine the association between the 2020 presidential elections and cardiovascular outcomes.

### Limitations

We also acknowledge that this study had some limitations. Results may not be fully generalizable to individuals in less integrated health care systems, uninsured individuals, or those in other regions of the country; this emphasizes the importance of reproducibility in other populations. Furthermore, we do not have measures of stress captured in the electronic health record and cannot make direct connections between political stress, individual anxiety, and acute CVD risk. Variability by place of residence and political affiliation was not examined; however, acute CVD risk can be influenced by both negative and positive emotional responses. Seasonal effects due to daylight savings time are associated with a higher risk of CVD events,^33^ therefore we cannot rule out the potential effect on our results. However, confounding is of minimal concern given the short time windows chosen for event identification in the current analysis. In our prior study, we did not find higher rates of CVD events in the non–election years 2015 and 2017 comparing the same time windows used for the 2016 election.^6^

## Conclusions

Higher rates of acute CVD were observed in the 5 days following the 2020 presidential election compared with the same days of the week before the election, confirming findings from the 2016 election in a larger population and with additional end points. There is a need for awareness of this higher risk of CVD, and further research is warranted to identify strategies that mitigate cardiovascular risk during notable political events.

## Notes

Supplement. **eTable 1.** Hospitalization for Acute Cardiovascular Disease (CVD) Events in the 5 Days Following the 2020 Presidential Election to the Same Days of the Week Before the Election, by KP Region. **eTable 2.** Hospitalization for Acute Cardiovascular Disease (CVD) Events in the 5 Days Following the 2020 Presidential Election (November 4-8) to the Same Days of the Week Two and Three Weeks Before the Election (October 14-18 and 21-25) **eTable 3.** Hospitalization for Acute Myocardial Infarction, Stroke, and Heart Failure in the 5 Days Following the 2020 Presidential Election (November 4-8) to the Same Days of the Week Two and Three Weeks Before the Election (October 14-18 and 21-25) **eTable 4.** Hospitalization for Acute Cardiovascular Disease (CVD) Events in the 5 Days Following the 2020 Presidential Election to the Same Days of the Week Before the Election–Excluding Patients with COVID-19 During the Same Encounter **eTable 5.** Hospitalization for Acute Myocardial Infarction, Stroke, and Heart Failure Following the 2020 Presidential Election Compared to the Same Days of the Week Before The Election–Excluding Patients With COVID-19 During the Same Encounter

## References

[1] Mittleman MA, Maclure M, Sherwood JB, ; Determinants of Myocardial Infarction Onset Study Investigators. Triggering of acute myocardial infarction onset by episodes of anger. Circulation. 1995;92(7):1720-1725. doi:10.1161/01.CIR.92.7.1720 7671353

[2] Mittleman MA, Mostofsky E. Physical, psychological and chemical triggers of acute cardiovascular events: preventive strategies. Circulation. 2011;124(3):346-354. doi:10.1161/CIRCULATIONAHA.110.968776 21768552 PMC3139921

[3] Leor J, Poole WK, Kloner RA. Sudden cardiac death triggered by an earthquake. N Engl J Med. 1996;334(7):413-419. doi:10.1056/NEJM199602153340701 8552142

[4] American Psychological Association. Stress in America: coping with change. part 1. Stress in America^TM^ Survey. Published 2017. Accessed March 18, 2022. https://www.apa.org/news/press/releases/stress/2016/coping-with-change.pdf

[5] American Psychological Association. Stress in America™ 2020: a national mental health crisis. Published 2020. Accessed March 18, 2022. https://www.apa.org/news/press/releases/stress/2020/report-october

[6] Mefford MT, Mittleman MA, Li BH, . Sociopolitical stress and acute cardiovascular disease hospitalizations around the 2016 presidential election. Proc Natl Acad Sci U S A. 2020;117(43):27054-27058. doi:10.1073/pnas.2012096117 33046627 PMC7604431

[7] Rosman L, Salmoirago-Blotcher E, Mahmood R, . Arrhythmia risk during the 2016 US presidential election: the cost of stressful politics. J Am Heart Assoc. 2021;10(11):e020559. doi:10.1161/JAHA.120.020559 34014121 PMC8483504

[8] Gemmill A, Catalano R, Casey JA, . Association of preterm births among US Latina women with the 2016 presidential election. JAMA Netw Open. 2019;2(7):e197084. doi:10.1001/jamanetworkopen.2019.7084 31322687 PMC6647358

[9] Krieger N, Huynh M, Li W, Waterman PD, Van Wye G. Severe sociopolitical stressors and preterm births in New York City: 1 September 2015 to 31 August 2017. J Epidemiol Community Health. 2018;72(12):1147-1152. doi:10.1136/jech-2018-211077 30327451 PMC6252370

[10] Koebnick C, Langer-Gould AM, Gould MK, . Sociodemographic characteristics of members of a large, integrated health care system: comparison with US Census Bureau data. Perm J. 2012;16(3):37-41. doi:10.7812/TPP/12-031 23012597 PMC3442759

[11] Gordon N, Lin T. The Kaiser Permanente Northern California Adult Member Health Survey. Perm J. 2016;20(4):15-225. doi:10.7812/TPP/15-225 27548806 PMC5101088

[12] von Elm E, Altman DG, Egger M, Pocock SJ, Gøtzsche PC, Vandenbroucke JP; STROBE Initiative. The Strengthening the Reporting of Observational Studies in Epidemiology (STROBE) statement: guidelines for reporting observational studies. Prev Med. 2007;45(4):247-251. doi:10.1016/j.ypmed.2007.08.012 17950122

[13] Kokotailo RA, Hill MD. Coding of stroke and stroke risk factors using international classification of diseases, revisions 9 and 10. Stroke. 2005;36(8):1776-1781. doi:10.1161/01.STR.0000174293.17959.a1 16020772

[14] Go AS, Lee WY, Yang J, Lo JC, Gurwitz JH. Statin therapy and risks for death and hospitalization in chronic heart failure. JAMA. 2006;296(17):2105-2111. doi:10.1001/jama.296.17.2105 17077375

[15] Levine GN, Cohen BE, Commodore-Mensah Y, . Psychological health, well-being, and the mind-heart-body connection: a scientific statement from the American Heart Association. Circulation. 2021;143(10):e763-e783. doi:10.1161/CIR.0000000000000947 33486973

[16] Chida Y, Steptoe A. The association of anger and hostility with future coronary heart disease: a meta-analytic review of prospective evidence. J Am Coll Cardiol. 2009;53(11):936-946. doi:10.1016/j.jacc.2008.11.044 19281923

[17] Meyer GJ, Katko NJ, Mihura JL, Klag MJ, Meoni LA. The incremental validity of self-report and performance-based methods for assessing hostility to predict cardiovascular disease in physicians. J Pers Assess. 2018;100(1):68-83. doi:10.1080/00223891.2017.1306780 28418719

[18] Harris KM, Gottdiener JS, Gottlieb SS, Burg MM, Li S, Krantz DS. Impact of mental stress and anger on indices of diastolic function in patients with heart failure. J Card Fail. 2020;26(11):1006-1010. doi:10.1016/j.cardfail.2020.07.008 32750485 PMC7704726

[19] Emdin CA, Odutayo A, Wong CX, Tran J, Hsiao AJ, Hunn BH. Meta-analysis of anxiety as a risk factor for cardiovascular disease. Am J Cardiol. 2016;118(4):511-519. doi:10.1016/j.amjcard.2016.05.041 27324160

[20] Suls J. Anger and the heart: perspectives on cardiac risk, mechanisms and interventions. Prog Cardiovasc Dis. 2013;55(6):538-547. doi:10.1016/j.pcad.2013.03.002 23621963

[21] Cohen BE, Edmondson D, Kronish IM. State of the art review: depression, stress, anxiety, and cardiovascular disease. Am J Hypertens. 2015;28(11):1295-1302. doi:10.1093/ajh/hpv047 25911639 PMC4612342

[22] Goldbacher EM, Matthews KA. Are psychological characteristics related to risk of the metabolic syndrome? A review of the literature. Ann Behav Med. 2007;34(3):240-252. doi:10.1007/BF02874549 18020934

[23] Tindle HA, Duncan MS, Liu S, . Optimism, pessimism, cynical hostility, and biomarkers of metabolic function in the Women’s Health Initiative. J Diabetes. 2018;10(6):512-523. doi:10.1111/1753-0407.12584 28703425 PMC8835287

[24] Sumner JA, Nishimi KM, Koenen KC, Roberts AL, Kubzansky LD. Posttraumatic stress disorder and inflammation: untangling issues of bidirectionality. Biol Psychiatry. 2020;87(10):885-897. doi:10.1016/j.biopsych.2019.11.005 31932029 PMC7211139

[25] Lampert R. Behavioral influences on cardiac arrhythmias. Trends Cardiovasc Med. 2016;26(1):68-77. doi:10.1016/j.tcm.2015.04.008 25983071 PMC4609244

[26] Gullette EC, Blumenthal JA, Babyak M, . Effects of mental stress on myocardial ischemia during daily life. JAMA. 1997;277(19):1521-1526. doi:10.1001/jama.1997.03540430033029 9153365

[27] American Psychological Association. Stress in America 2012: stress and gender. 2012. Accessed December 16, 2021. https://www.apa.org/news/press/releases/stress/2012/gender

[28] Soumya RS, Unni TG, Raghu KG. Impact of COVID-19 on the cardiovascular system: a review of available reports. Cardiovasc Drugs Ther. 2021;35(3):411-425. doi:10.1007/s10557-020-07073-y 32926272 PMC7487338

[29] Greenwood BN, Fleshner M. Exercise, stress resistance, and central serotonergic systems. Exerc Sport Sci Rev. 2011;39(3):140-149. doi:10.1097/JES.0b013e31821f7e45 21508844 PMC4303035

[30] Nijjar PS, Connett JE, Lindquist R, . Randomized trial of mindfulness-based stress reduction in cardiac patients eligible for cardiac rehabilitation. Sci Rep. 2019;9(1):18415. doi:10.1038/s41598-019-54932-2 31804580 PMC6895078

[31] Pascoe MC, Thompson DR, Ski CF. Yoga, mindfulness-based stress reduction and stress-related physiological measures: a meta-analysis. Psychoneuroendocrinology. 2017;86:152-168. doi:10.1016/j.psyneuen.2017.08.008 28963884

[32] Levine GN, Lange RA, Bairey-Merz CN, ; American Heart Association Council on Clinical Cardiology; Council on Cardiovascular and Stroke Nursing; and Council on Hypertension. Meditation and cardiovascular risk reduction: a scientific statement from the American Heart Association. J Am Heart Assoc. 2017;6(10):e002218. doi:10.1161/JAHA.117.002218 28963100 PMC5721815

[33] Manfredini R, Fabbian F, De Giorgi A, . Daylight saving time and myocardial infarction: should we be worried? a review of the evidence. Eur Rev Med Pharmacol Sci. 2018;22(3):750-755. 29461606 10.26355/eurrev_201802_14306

