# Supplementary material for: Association of the 2020 US Presidential Election With Hospitalizations for Acute Cardiovascular Conditions
Source: JAMA Netw Open. 2022 Apr 20;5(4):e228031. doi: 10.1001/jamanetworkopen.2022.8031 (PMC9021908; doi:10.1001/jamanetworkopen.2022.8031)
Supplement: Supplement. — eTable 1. Hospitalization for Acute Cardiovascular Disease (CVD) Events in the 5 Days Following the 2020 Presidential Election to the Same Days of the Week Before the Election, by KP Region. eTable 2. Hospitalization for Acute Cardiovascular Disease (CVD) Events in the 5 Days Following the 2020 Presidential Election (November 4-8) to the Same Days of the Week Two and Three Weeks Before the Election (October 14-18 and 21-25) eTable 3. Hospitalization for Acute Myocardial Infarction, Stroke, and Heart Failure in the 5 Days Following the 2020 Presidential Election (November 4-8) to the Same Days of the Week Two and Three Weeks Before the Election (October 14-18 and 21-25) eTable 4. Hospitalization for Acute Cardiovascular Disease (CVD) Events in the 5 Days Following the 2020 Presidential Election to the Same Days of the Week Before the Election–Excluding Patients with COVID-19 During the Same Encounter eTable 5. Hospitalization for Acute Myocardial Infarction, Stroke, and Heart Failure Following the 2020 Presidential Election Compared to the Same Days of the Week Before The Election–Excluding Patients With COVID-19 During the Same Encounter [file jamanetwopen-e228031-s001.pdf]

Supplemental Online Content

Mefford MT, Rana JS, Reynolds K, et al. Association of the 2020 US presidential election with hospitalizations for acute cardiovascular conditions. *JAMA Netw Open.* 2022;5(4):e228031. doi:10.1001/jamanetworkopen.2022.8031

eTable 1. Hospitalization for Acute Cardiovascular Disease (CVD) Events in the 5 Days Following the 2020 Presidential Election to the Same Days of the Week Before the Election, by KP Region.

eTable 2. Hospitalization for Acute Cardiovascular Disease (CVD) Events in the 5 Days Following the 2020 Presidential Election (November 4-8) to the Same Days of the Week Two and Three Weeks Before the Election (October 14-18 and 21-25)

eTable 3. Hospitalization for Acute Myocardial Infarction, Stroke, and Heart Failure in the 5 Days Following the 2020 Presidential Election (November 4-8) to the Same Days of the Week Two and Three Weeks Before the Election (October 14-18 and 21-25)

eTable 4. Hospitalization for Acute Cardiovascular Disease (CVD) Events in the 5 Days Following the 2020 Presidential Election to the Same Days of the Week Before the Election–Excluding Patients with COVID-19 During the Same Encounter

eTable 5. Hospitalization for Acute Myocardial Infarction, Stroke, and Heart Failure Following the 2020 Presidential Election Compared to the Same Days of the Week Before The Election–Excluding Patients With COVID-19 During the Same Encounter

This supplemental material has been provided by the authors to give readers additional information about their work.

© 2022 Mefford MT et al. *JAMA Network Open.*

eTable 1. Hospitalization for acute cardiovascular disease (CVD) events in the 5 days following the 2020 presidential election to the same days of the week before the election, by KP region.

	November 4-8 (Risk Window)				October 21-25 (Control Window)			Rate Ratio (95% CI)	P-Value
	**Events, N**	**Person-Years**	**Rate per 100,000 person-years**		**Events, N**	**Person-Years**	**Rate per 100,000 person-years**		
**All CVD**									
**KPSC**	389	44,357.19	876.972		312	44,469.78	701.600	1.25 (1.08, 1.45)	0.207
**KPNC**	277	43,220.63	640.900		257	43,342.68	592.950	1.08 (0.91,1.28)	
**AMI**									
**KPSC**	96	44,357.19	216.425		63	44,469.78	141.669	1.53 (1.11, 2.10)	0.533
**KPNC**	83	43,220.63	192.040		63	43,342.68	145.35	1.32 (0.95,1.83)	
**Stroke**									
**KPSC**	176	44,357.19	396.779		154	44,469.78	346.303	1.14 (0.92, 1.42)	0.079
**KPNC**	89	43,220.63	205.920		107	43,342.68	246.87	0.83 (0.63,1.10)	
**HF**									
**KPSC**	130	44,357.19	293.075		109	44,469.78	245.110	1.19 (0.93, 1.54)	0.884
**KPNC**	109	43,220.63	252.190		94	43,342.68	216.880	1.16 (0.88,1.53)	

Abbreviations: AMI, acute myocardial infarction; CI, confidence interval; CVD, cardiovascular disease; HF, heart failure; KPNC, Kaiser Permanente Northern California; KPSC, Kaiser Permanente Southern California

© 2022 Mefford MT et al. *JAMA Network Open.*

eTable 2. Hospitalization for acute cardiovascular disease (CVD) events in the 5 days following the 2020 presidential election (November 4-8) to the same days of the week two and three weeks before the election (October 14-18 and 21-25).

	**November 4-8 (Risk Window)**			**October 14-18 & 21-25 (Control Window)**			**Rate Ratio (95% CI)**	**P-value**
	**Events, N**	**Person-Years**	**Rate per 100,000 person-years**	**Events, N**	**Person-Years**	**Rate per 100,000 person-years**		
**Overall**	666	87,577.82	760.47	1,132	175,599.13	644.65	1.18 (1.09, 1.30)	
**Age, years**								
18-54	87	54,345.96	160.09	139	109,068.75	127.44	1.26 (0.96, 1.64)	0.32
55-74	278	25,964.30	1,070.7	514	51,988.20	988.69	1.08 (0.94, 1.25)	
75+	301	7,267.56	4,141.69	479	14,542.18	3,293.87	1.26 (1.09, 1.45)	
**Sex**								
Male	388	40,721.97	952.80	606	81,684.27	741.88	1.28 (1.13, 1.46)	0.05
Female	278	46,855.85	593.31	526	93,914.86	560.08	1.05 (0.92, 1.23)	
**Race/Ethnicity**								
Asian/Pacific Islander	87	14,828.56	586.71	155	29,703.92	521.82	1.12 (0.86, 1.46)	0.80
Black	89	6,922.46	1,285.67	157	13,877.62	1,131.32	1.14 (0.88, 1.47)	
Hispanic	150	28,771.25	521.35	270	57,641.41	468.41	1.11 (0.92, 1.36)	
Other	4	887.27	450.82	10	1,779.20	562.05	--	
White	336	36,168.29	928.99	540	72,596.97	743.83	1.25 (1.09, 1.43)	

Abbreviation: CI, confidence interval

*Other race/ethnicity includes Native American Alaskan, multiple ethnicities, and those not specified as above.

© 2022 Mefford MT et al. *JAMA Network Open.*

eTable 3. Hospitalization for acute myocardial infarction, stroke, and heart failure in the 5 days following the 2020 presidential election (November 4-8) to the same days of the week two and three weeks before the election (October 14-18 and 21-25).

	**November 4-8 (Risk Window)**			**October 14-18 & 21-25 (Control Window)**			**Rate Ratio (95% CI)**
	**Events, N**	**Person-Years**	**Rate per 100,000 person-years**	**Events, N**	**Person-Years**	**Rate per 100,000 person-years**	
**AMI**	179	87,577.82	204.39	279	175,599.13	158.89	1.29 (1.07, 1.55)
**Stroke**	265	87,577.82	302.59	500	175,599.13	284.74	1.06 (0.92, 1.23)
**Heart Failure**	239	87,577.82	272.90	383	175,599.13	218.11	1.25 (1.06, 1.47)

Abbreviation: AMI, acute myocardial infarction; CI, confidence interval

© 2022 Mefford MT et al. *JAMA Network Open.*

eTable 4. Hospitalization for acute cardiovascular disease (CVD) events in the 5 days following the 2020 presidential election to the same days of the week before the election– excluding patients with COVID-19 during the same encounter.

	**November 4-8 (Risk Window)**			**October 21-25 (Control Window)**			**Rate Ratio (95% CI)**	**P-value**
	**Events, N**	**Person-Years**	**Rate per 100,000 person-years**	**Events, N**	**Person-Years**	**Rate per 100,000 person-years**		
**Overall**	654	87,577.82	746.76	561	87,812.46	638.86	1.17 (1.04, 1.31)	
**Age, years**								
18-54	85	54,345.96	156.41	67	54,533.92	122.86	1.27 (0.92, 1.75)	0.11
55-74	273	25,964.30	1051.44	268	26,000.44	1,030.75	1.02 (0.86, 1.21)	
75+	296	7,267.56	4,072.89	226	7,278.1	3,105.21	1.31 (1.10, 1.56)	
**Sex**								
Male	382	40,721.97	938.07	295	40,848.65	722.18	1.30 (1.12, 1.51)	0.04
Female	272	46,855.85	580.50	266	46,963.81	566.39	1.02 (0.87, 1.21)	
**Race/Ethnicity**								
Asian/Pacific Islander	85	14,828.56	573.22	84	14,854.03	565.50	1.01 (0.74, 1.37)	0.53
Black	88	6,922.46	1,271.23	83	6,939.75	1,196.01	1.06 (0.79, 1.44)	
Hispanic	146	28,771.25	507.45	128	28,826.69	444.03	1.14 (0.90, 1.45)	
Other	4	887.27	450.82	6	889.91	674.23	--	
White	331	36,168.29	915.17	260	36,302.09	716.21	1.28 (1.09, 1.50)	

Abbreviation: CI, confidence interval

*Other race/ethnicity includes Native American Alaskan, multiple ethnicities, and those not specified as above.

© 2022 Mefford MT et al. *JAMA Network Open.*

eTable 5. Hospitalization for acute myocardial infarction, stroke, and heart failure following the 2020 presidential election compared to the same days of the week before the election– excluding patients with COVID-19 during the same encounter.

	**November 4-8 (Risk Window)**			**October 21-25 (Control Window)**			**Rate Ratio (95% CI)**
	**Events, N**	**Person-Years**	**Rate per 100,000 person-years**	**Events, N**	**Person-Years**	**Rate per 100,000 person-years**	
**AMI**	177	87,577.82	202.106	123	87,812.46	140.071	1.44 (1.15, 1.82)
**Stroke**	257	87,577.82	293.453	256	87,812.46	291.53	1.01 (0.85, 1.20)
**Heart Failure**	237	87,577.82	270.616	202	87,812.46	230.036	1.18 (0.98, 1.42)

Abbreviation: AMI, acute myocardial infarction; CI, confidence interval

© 2022 Mefford MT et al. *JAMA Network Open.*
